# Development of a Novel Model for the Assessment of Dead-Space Management in Soft Tissue

**DOI:** 10.1371/journal.pone.0136514

**Published:** 2015-08-25

**Authors:** Rema A. Oliver, Vedran Lovric, Yan Yu, Chris Christou, Sean S. Aiken, John J. Cooper, William R. Walsh

**Affiliations:** 1 Surgical & Orthopaedic Research Laboratories, University of New South Wales Australia, Sydney, Australia, Prince of Wales Clinical School, Prince of Wales Hospital, Randwick, NSW, Australia; 2 Biocomposites Ltd., Keele Science Park, Staffordshire, United Kingdom; Chang Gung University, TAIWAN

## Abstract

Following extensive surgical debridement in the treatment of infection, a “dead space” can result following surgical closure that can fill with hematoma, an environment conducive to bacterial growth. The eradication of dead space is essential in order to prevent recurrent infection. This study describes a novel small animal model to investigate dead-space management in muscle tissue. Two absorbable test materials were implanted in each animal; beads of calcium sulfate alone, and beads loaded with vancomycin and tobramycin. In-life blood samples and radiographs were taken from each animal following implantation. Animals were sacrificed at 1, 7, 21, 42, and 63 days post-operatively (n = 4), and implant sites were analysed by micro-computed tomography, histology and immunohistochemistry. Complete resorption was confirmed radiographically at 3 weeks post-implantation. Histologically, the host tissue response to both materials was identical, and subsequent healing at the implant sites was observed with no dead space remaining. Vancomycin was not detected in blood serum. However, peak tobramycin levels were detected in all animals at 6 hours post-implantation with no detectable levels in any animals at 72 hours post implantation. Serological inflammatory cytokine expression for IL-6, TNF-α and IL-1β indicated no unusual inflammatory response to the implanted materials or surgical procedure. The model was found to be convenient and effective for the assessment of implant materials for management of dead space in muscle tissue. The two materials tested were effective in resolving the surgically created dead space, and did not elicit any unexpected adverse host response.

## Introduction

When extensive debridement is required, patients are left with a dead space that, when closed, can fill with a hematoma or seroma, an environment conducive to the growth of bacteria. Clinical results suggest that the major factor for successful eradication of infection is surgical debridement, dead space management [1] and prevention of bacterial colonization. The filling of dead space has been considered essential clinical practice in septic surgery for over 60 years [2].

Local tissue flaps or free flaps may be used to fill dead space [3, 4]. However, this is not always possible, especially if significant debridement of soft tissue has taken place in the surgical site due to extensive removal of infected tissues. Antibiotic-impregnated polymethylmethacrylate (PMMA) beads impregnated with antibiotics, have been used for over three decades [5]. These beads are commonly used inside dead space [6, 7]. PMMA is frequently used in joint revision surgery in the form of a spacer [8, 9], and in the management of infected fractures [10] or in the form of beads on a wire or as a pouch [11].

PMMA may have some disadvantages. As a non-resorbing material, in many circumstances it must be removed in an additional surgical procedure when its function has been completed, due to the risk of becoming a nidus for future infection [12, 13]. Also, a fibrin layer may form around PMMA beads following implantation, which can also serve as an additional nidus for infection [14]. Laboratory studies show that bacteria can readily attach and form biofilms on PMMA within four days [15].

The use of alternative materials has been reported. Mackey et al reported results with antibiotic with calcium sulfate beads, adding them to reduce the dead space remaining following surgery, therefore reducing post-operative hematoma formation. The authors believed that this reduction in hematoma formation, a potential culture medium for any remaining bacteria, possibly explained the positive outcomes when using calcium sulfate in the treatment of osteomyelitis cavities. They also reported that when used in the presence of a draining wound, calcium sulfate is either resorbed or drains in the pus, and does not remain as a sequestrum [16].

The technique of transmetatarsal amputation has also described the placement of antibiotic-impregnated calcium sulfate beads with careful attention to soft-tissue technique, placing them into the dead space between the skin flaps. This was reported to prevent recontamination of the deep space should wound necrosis occur [17].

A logical decision regarding whatever filler is used, is the need for some kind of protection against colonisation with pathogenic bacteria [18]. Device-related infections (DRI’s) are a significant issue in implant surgery as well as with short term biomedical devices [19]. Indeed, microbial infections resulting from bacterial adhesion to biomaterial surfaces have been observed on almost all medical devices [20]. These are frequently observed as biofilm infections on the implant, and pose a number of clinical challenges due to their resistance to immune defence mechanisms and antimicrobials. In order to reduce the incidence of device related infections, the incorporation of antibiotics into devices has been explored by a number of researchers, using a variety of materials. These materials have been the subject of a number of reviews [19–23].

Animal models have been used previously to demonstrate the efficacy of materials for dead-space management and tissue regeneration in bone tissue [24–26]. However, no animal model has been reported to investigate the safety and efficacy of implant materials for dead-space management in muscle tissue. This study describes a novel animal model to investigate dead-space management in soft tissue, and the assessment of calcium sulfate in this model, alone and in combination with two antibiotics, vancomycin and tobramycin.

## Materials and Methods

Implant materials (1 cc per site, 5 beads of material) were placed into intramuscular sites following retraction of the longissimus muscles at 4 non adjacent implant sites in 24 adult New Zealand white rabbits to mimic implantation for dead-space management in muscle tissue following approval from the Animal Care and Ethics Committee of the University of New South Wales (ACEC #: 13/67A). Two test materials were implanted; beads of calcium sulfate alone (CS), and beads of calcium sulfate combined with vancomycin and tobramycin (CSVT).

### Preparation of implant materials

Under sterile conditions, 10 mL kits containing 20g of pharmaceutical grade calcium sulfate alpha-hemihydrate powder (Stimulan, Biocomposites Ltd, U.K.) were used to prepare the beads for implantation; 20g of calcium sulfate alpha-hemihydrate was mixed with 6 mL of the provided mixing solution for unloaded beads. For the antibiotic containing beads, 20g of calcium sulfate alpha-hemihydrate was mixed with 1000 mg vancomycin hydrochloride powder (Hospira UK Limited) and thoroughly blended prior to the addition of 6 mL of tobramycin sulfate solution (40 mg/mL, Hospira UK Limited).

**Fig 1:**
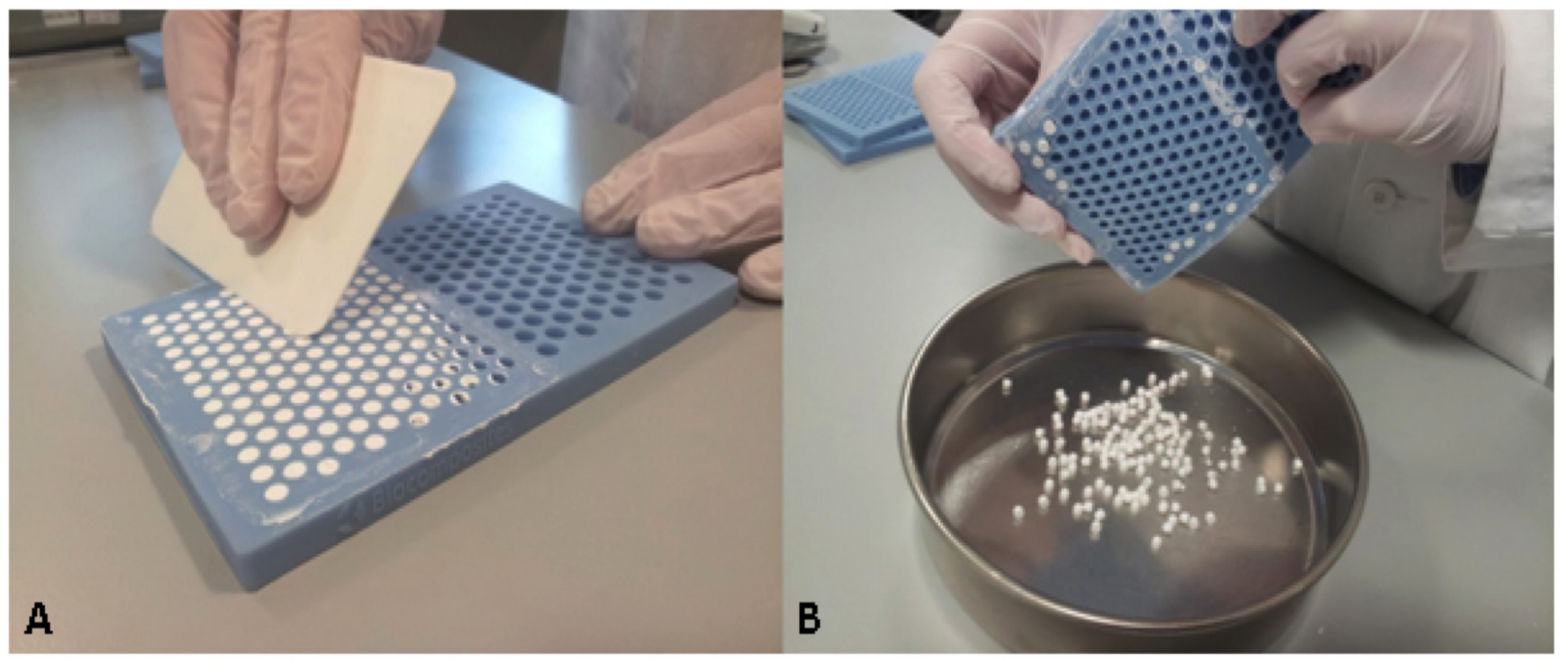
Preparation of implant material. A. Preparation of the calcium sulfate beads using the flexible mould. B. Removal of beads from mould once set hard.

In each case, all components were mixed thoroughly for 30 to 60 seconds to form a smooth paste which was pressed into 6.0mm diameter hemispherical cavities in the flexible mould (Fig 1A). The beads were left undisturbed for 30 to 60 minutes to hydrate and set according to the reaction:

$${\text{CaSO}}_{\text{4}}\text{. }{\text{½H}}_{\text{2}}{\text{O + 1½H}}_{\text{2}}{\text{O = CaSO}}_{\text{4}}\text{. }{\text{2H}}_{\text{2}}\text{O}$$

When set hard the beads were removed by flexing the mould (Fig 1B).

**Fig 2:**
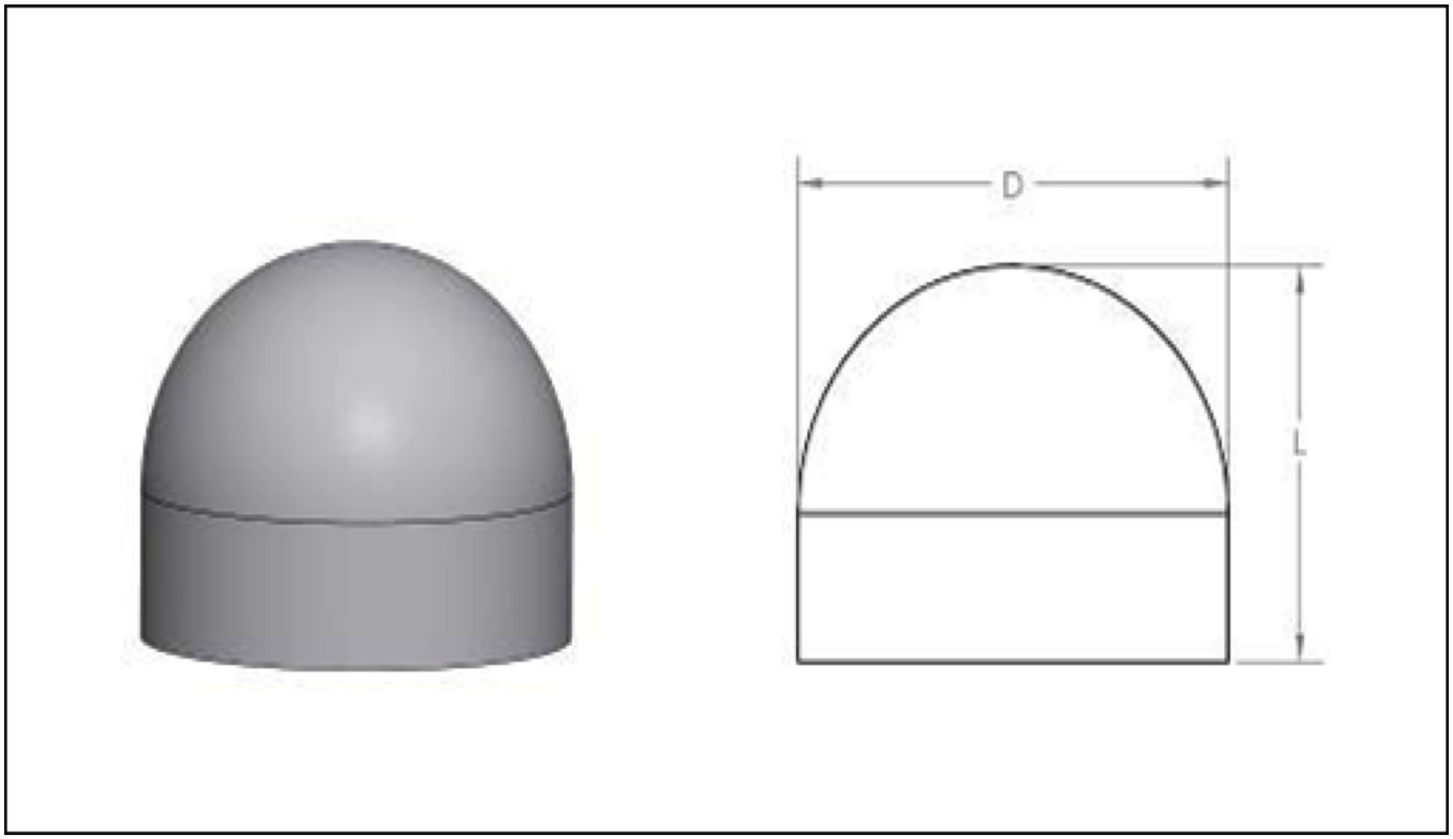
Shape of beads. Shape of beads produced by the flexible mould.

The beads were hemispherical with a diameter (D) of 6mm and a length (L) of 4.8mm (Fig 2).

A homogenous mixture of antibiotic and calcium sulfate alpha-hemihydrate was achieved as a result of thorough mixing in the preparation of the beads, enabling the calculation of concentration of antibiotic, as weight/weight of beads.

### Peripheral blood analyses

Peripheral blood was taken pre-operatively, 1, 6, 12, 24 hours and 3, 7, 14, 21, 42 and 63 days throughout the study for standard blood panel haematology/biochemistry (IDEXX Laboratories, Sydney, Australia), serum cytokine levels (IL-1β, IL-6 and TNF-α) and to determine systemic Vancomycin and Tobramycin levels.

Blood was collected into a Vacutainer Plain Clot 4mL tube and allowed to clot at room temperature for 2 hours before centrifugation for 15 minutes at 2000xg. The serum was carefully removed and stored at -80°C until needed.

Serum IL-1β (E04I0010), IL-6 (CSB-E06903Rb) and TNF-α (CSB-E06998Rb) levels were analysed by enzyme-linked immunosorbent assay (ELISA) using commercially available kits (Cusabio Biotech, Beijing, China) according to the manufacturer’s instructions. Each sample was measured in duplicate and standards were also run on each 96-well plate. The detection limits within serum for each of these tested cytokines are 1 pg/ml, 15.6 pg/ml and 78 pg/ml respectively.

### Surgery

**Fig 3:**
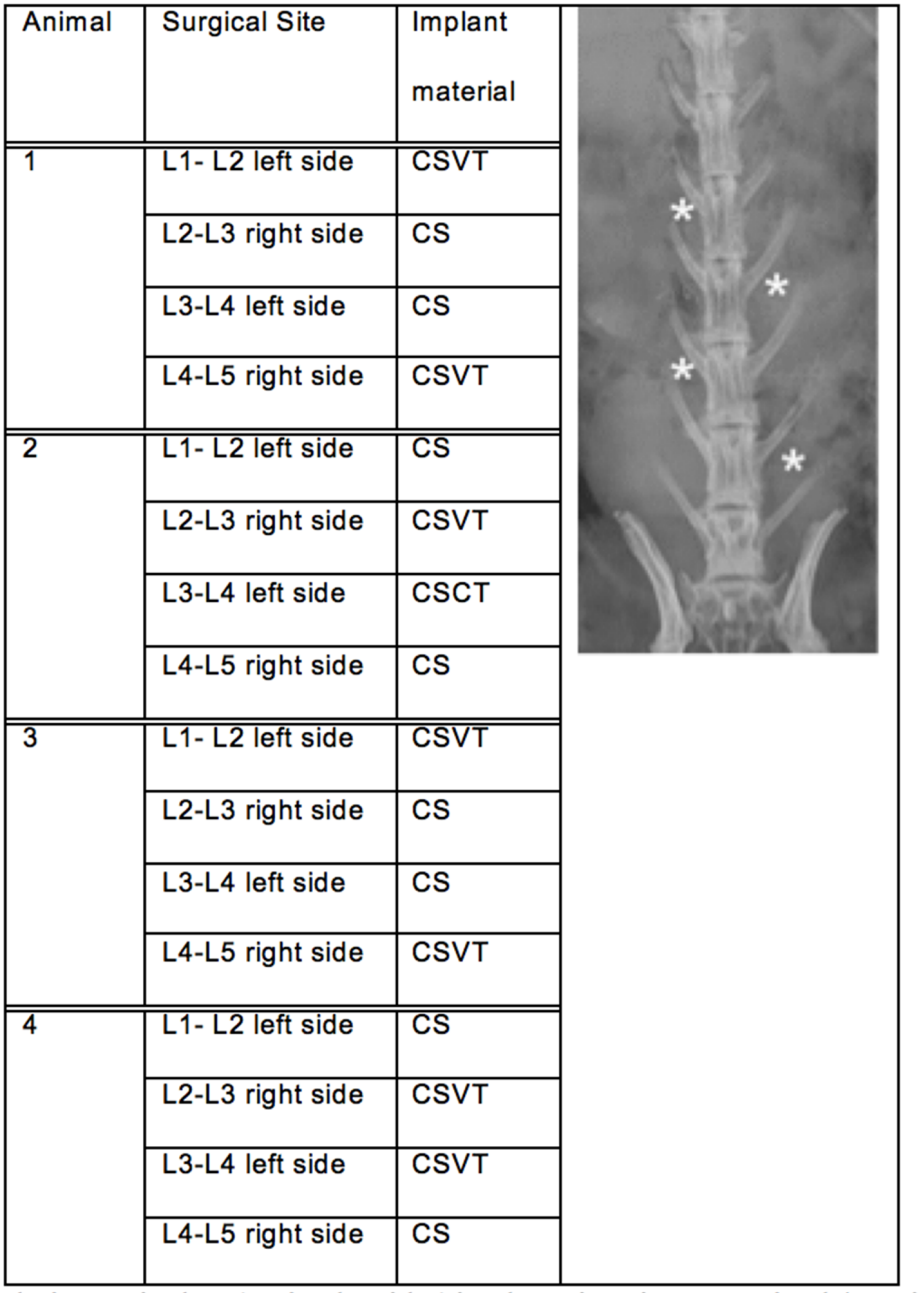
Study design and implantation schematic. Each time point has 4 animals with 4 implantation sites per animal (astericks).

**Fig 4:**
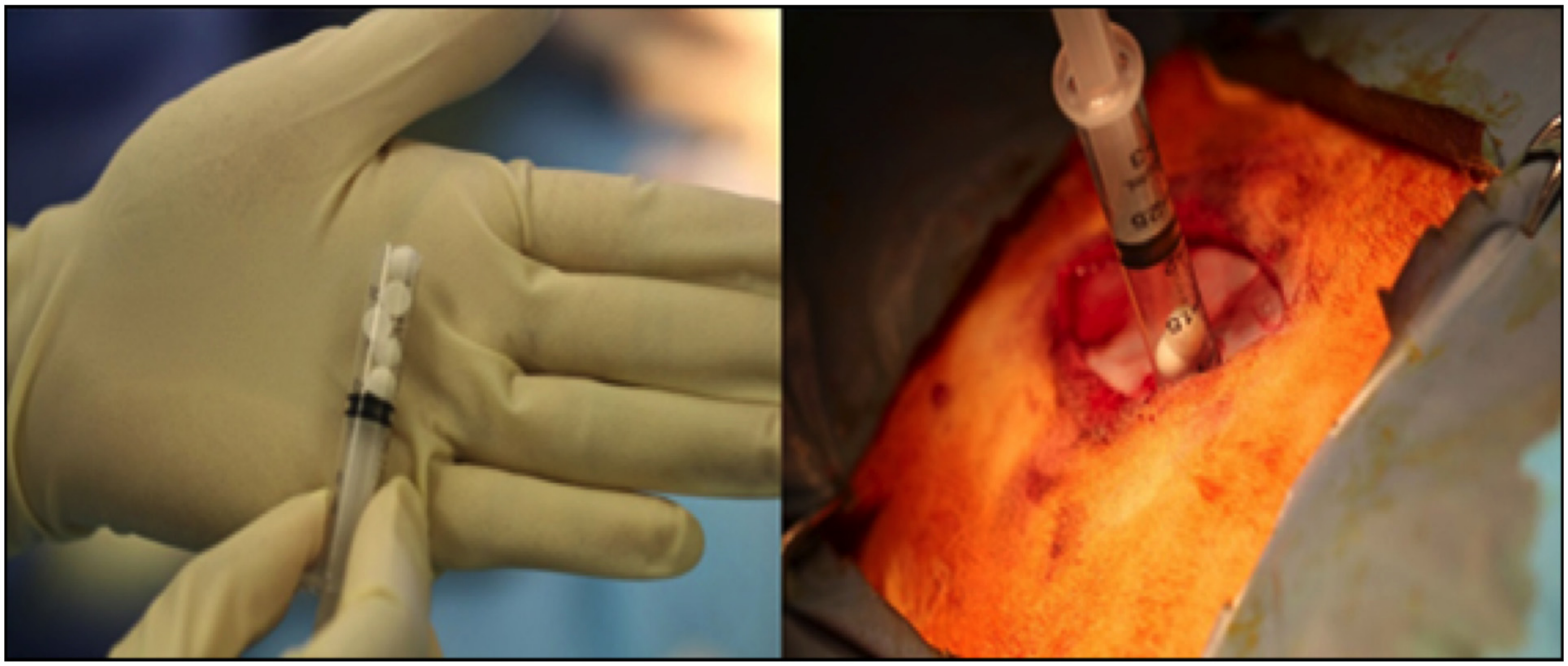
Implantation of beads. Beads were implanted into individual implantation sites using pre-packed syringes, modified with the tip removed.

For all 24 animals in the study, four implant sites were used per animal, two sites each side of the spine, in non-adjacent intramuscular sites (longissimus muscles) above the levels in L1-L2, L2-L3, L3-L4 and L4-L5 (Fig 3). The intermuscular plane between the multifidus and longissimus muscles was retracted to create a 1cm x 2cm void. The void was filled with approximately 1cc of test material, representing five beads of material per implant site. The beads were allocated in a sterile fashion into sterile 3cc syringes with the tip removed to facilitate implantation (Fig 4). This allowed uniform implantation and reproducible implantation. Each animal received CS beads in two sites and CSVT beads in the remaining two. The fascial incisions were closed with an individual single strand non-absorbable suture. Closure was achieved with equidistant adjacent stitches at approximately 3mm intervals. The skin of the incision was closed with an individual single strand absorbable suture in a continuous fashion. Animals were sacrificed in groups of four at 0, 1, 7, 21, 42, and 63 days post operatively.

For each sacrifice time point in the study the allocation of material to implant sites is as outlined in Fig 3.

Posteroanterior radiographs were used to visualise the appearance of the beads in the soft tissue at the time of surgery. Radiographs were taken with a mobile xray machine (Poskom Co., Ltd, Korea) and digital plates (AGFA, Sydney Australia). These images were used as a baseline for examination at later time points. During the study, in life blood samples and in life postero-anterior and lateral radiographs were taken from each animal following implantation. The levels of vancomycin and tobramycin in the blood serum samples versus time are also reported. The *in-vivo* absorption of the test materials was quantified by analysis of standard radiographs and micro-computed tomography (μCT).

### Euthanasia and necropsy

After the scheduled duration was complete, the animals were euthanized, radiographs and μCT was performed, and the implant sites were inspected and harvested for evaluations to assess healing, local tissue reactions and local vancomycin and tobramycin levels.

Animals were sacrificed according to the study design: each time point had 4 animals with 4 implantation sites per animal. The *in-vivo* release kinetics of vancomycin and tobramycin in local tissues as well as serum levels were evaluated. The general integrity of the skin incision was noted along with the macroscopic reaction of the underlining subcutaneous tissues as normal or abnormal. If abnormal this was further assessed for evidence of infection or macroscopic signs of inflammation/foreign body reaction.

At the time of harvest the heart, liver, kidney, spleen, and lungs were examined and any abnormalities noted, for each animal. A portion of the distant organs was processed for routine paraffin histology and evaluated in a blinded fashion for any abnormalities. Microscopic changes were graded on a severity grade based upon the following: 1 = minimal, 2 = mild, 3 = moderate, 4 = severe, N = normal.

### Radiography

The animals were radiographed in the same manner as performed at the completion of surgery. The images were used to determine resorption based on radiographic appearance. Radiographic status of the implant sites was compared to radiographs obtained immediately after implantation to determine absorption of the implant material. The radiographs were graded in a blinded fashion for implant absorption on a scale of 0–4 (Table 1). Data was analysed using a non-parametric analysis of variance (Kruskal Wallis test) [27].

**Table 1:** Radiographic grading scale.

Radiographic grading	
Grade	Description: % Implant remaining
0	81–100
1	61–80
2	41–60
3	21–40
4	0–20

Radiographs were graded for implant resorption on a scale of 0–4.

### Micro-Computed Tomography

Micro-computed tomography (μCT) was performed for all animals using an *in-vivo* scanner (Inveon, Siemens Medical, PA, USA) in order to obtain high resolution images of the implant absorption. The surgical sites were scanned and the raw images reconstructed resulting in effective pixel size of 53.12 μm. 3D models were created using Siemens image analysis software (Inveon Research Workplace 3.0, Siemens Medical, PA, USA) to assess *in-vivo* absorption as well as any adverse tissue reactions. Images were examined in the axial, sagittal and coronal planes. The resultant images were graded in a blinded fashion for implant absorption on a scale of 0–4 (Table 1). Data was analysed using a non-parametric analysis of variance (Kruskal Wallis test) [27].

### Tissue sample harvesting and Vancomycin / Tobramycin levels

#### Residual CS and CSVT beads

The surgical sites were carefully dissected and examined for the presence of any residual beads. A portion of any residual beads present at the surgical sites was harvested. This material was allowed to air dry and placed in a desiccator for 24 hours and then morselized using a mortar and pestle. 0.1g of the powder was immersed in 1ml of serum for 24 hours. Vancomycin and tobramycin levels in the samples per gram of material were determined using a standard antibody assay.

#### Muscle tissue harvesting

A muscle sample (1x1cm) from the implantation site was harvested, air dried for 72 hours and then morselised using a mortar and pestle. 0.1g of the morselised tissue was immersed in 1ml of serum for 24 hours. The local concentration of vancomycin and tobramycin in the muscle sample was measured using a standard antibody assay.

#### Method validation; Rabbit serum Vancomycin and Tobramycin levels–in-vitro

Rabbit serum was harvested from rabbits from other studies at the time of sacrifice to perform a dose response curve for rabbit serum with a known concentration of vancomycin and tobramycin. Standard vancomycin and tobramycin levels in saline were also run for comparison. Concentration levels of 0.1, 1, 5, 10, 20, 50, 100, 200, 500 and 1000 μg/ml were determined to provide a standard curve. This portion of the study was completed with a standardised assay (Roche Cobas 6000, Roche Diagnostics, Mannheim, Germany). The assay method was based on a homogeneous enzyme immunoassay technique used for the quantitative analysis of antibiotic in human serum or plasma. (Vancomycin-Assay range: 1.7–80 mg/L, Assay precision: Total CV < 5%, Tobramycin Assay range: 0.33–10 mg/L, Assay precision: Total CV < 5%).

The serum samples collected post implantation were then analysed using this assay.

### Histology

Harvested implant sites allocated for histology were immediately fixed in phosphate buffered formalin for a minimum of 48 hours. They were then decalcified in 10% formic acid–phosphate buffered formalin at room temperature. The decalcified samples were placed into embedding blocks for paraffin processing. Paraffin blocks were sectioned to 5 microns using a microtome (Leica, Germany) and placed onto super-frosted slides for routine haematoxylin and eosin (H&E) staining. Stained sections were photographed using an Olympus Microscope (Olympus, Japan) and Olympus DP72 Camera. Histology was qualitatively assessed versus time at each time point. *In-vivo* response and biocompatibility to the materials was assessed at the implant site/ host tissue boundary.

### Immunohistochemistry

Immunohistochemistry was performed on paraffin sections from all animals at all sites for markers of inflammatory response including IL-1β, CD3, CD79a and IL-6 using standard techniques [28–32]. Paraffin sections were de-waxed in xylene and hydrated in a graded series of ethanol. A modified target retrieval citrate buffer, pH 6.1 (Dako Australia) was used for the recovery of antigenicity. Endogenous peroxidise was quenched with 0.3% hydrogen peroxide in 50% methanol for 10 minutes. Primary mouse monoclonal antibodies against IL-6 (ab9324, Abcam Plc, Cambridge, UK), IL-1β (MAB201, R&D Systems, Minneapolis MN, USA), CD79a (M7051, Dako Australia) and CD3 (55274, BD Biosciences, San Jose, CA USA) were incubated with the sections (one antibody per section) overnight at 4°C in humidity chambers. Non-immunised mouse IgG (Dako Australia) was used as a negative control. The final concentrations for the primary antibody and control was 0.5μg/ml for IL-6, 5μg/ml for IL-1β, CD3 and mouse IgG, and 1:25 for CD79a. After three washes with phosphate-buffered saline with 0.05% tween-20 (PBS-T), the sections were incubated with DakoCytomation EnVision+ System-HRP labelled polymer (conjugated with anti-mouse secondary antibody) for one hour at room temperature. After four separate washes with PBS-T, a DAKO liquid diaminobenzidine (DAB) substrate-chromogen system was applied to the sections and a brown end product was allowed to develop at the site of the target antigens. All sections were counterstained with Harris haematoxylin and mounted with EUKITT medium (Kindler GmbH & Co., Freiburg, Germany) and coverslipped. Images were evaluated in a blinded fashion for evidence of protein expression versus time.

## Results

Surgery was completed without any adverse events. All animals recovered well following surgery. Macroscopic reactions at the level of the surgical incisions were monitored for each animal. There was no evidence of wound breakdown at any time point. Bead absorption was observed at day 7 and day 21. A slight reaction was noted at day 21 in one site in one animal where there was elevation of the subcutaneous tissues but the overall reaction appeared normal. All subcutaneous sites were normal at day 42 and day 63 and the surgical implantation sites appeared well healed. The distant organs appeared normal in all animals.

### Blood serum Vancomycin and Tobramycin levels

**Fig 5:**
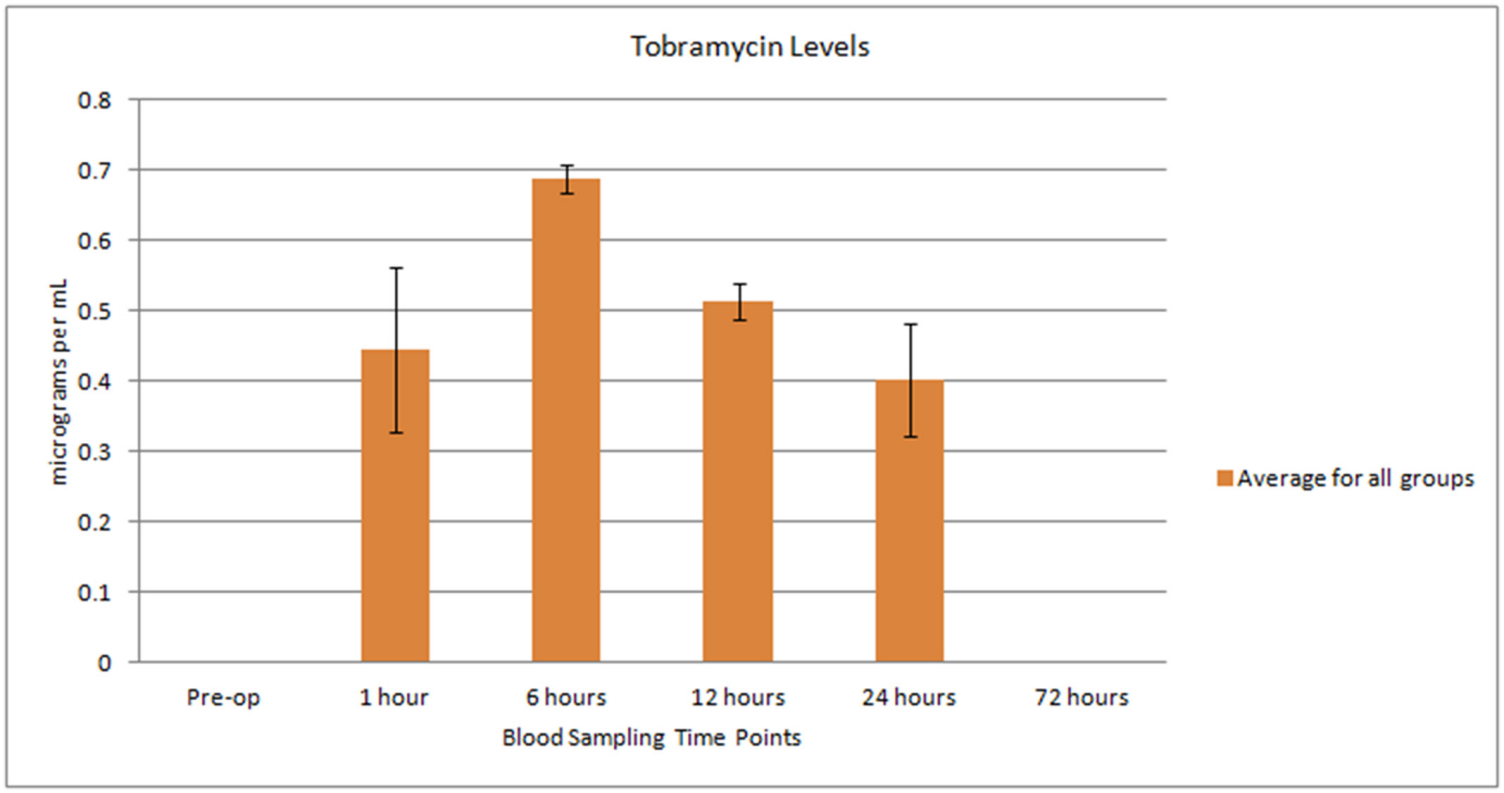
Graph indicating systemic tobramycin release. The average of all blood results available for testing at each of the predetermined blood sampling times for systemic tobramycin release.

Peripheral blood samples were collected prior to surgery (pre-op) and 1, 6, 12, and 24 hours following surgery as well as 3, 7, 14, 21, 42, and 63 days post implantation to determine the systemic serum vancomycin and tobramycin levels following implant material placement. Although the vancomycin assay was confirmed in preliminary validation of methodology using rabbit serum dilution experiments, no vancomycin was detected in blood serum in any animal at any time point in this study. Tobramycin levels were detected in 6 animals at 1 hour post implantation, following with detectable levels in all animals at 6 hours post implantation. The highest serum level detected at this time point was 0.88mg/L, with average values across all animals of 0.69mg/L. Serum levels of tobramycin then dropped in all subsequent time points for all animals, with detectable serum levels in only 4 animals at 24 hours post-implantation, and no detectable serum levels in any animals 72 hours post implantation (Fig 5).

### Blood serum inflammatory cytokine level (ELISA) analysis

By comparison with standard curves for each of the factors measured (IL-6, TNF-α and IL-1β), the levels of systemic cytokines were quantified preoperatively (baseline) and postoperatively at each of the allocated time points: 1hr, 6hrs, 12hrs, 24hrs, 72hrs, 7 days, 14 days, 21 days, 42 days and 63 days.

**Fig 6:**
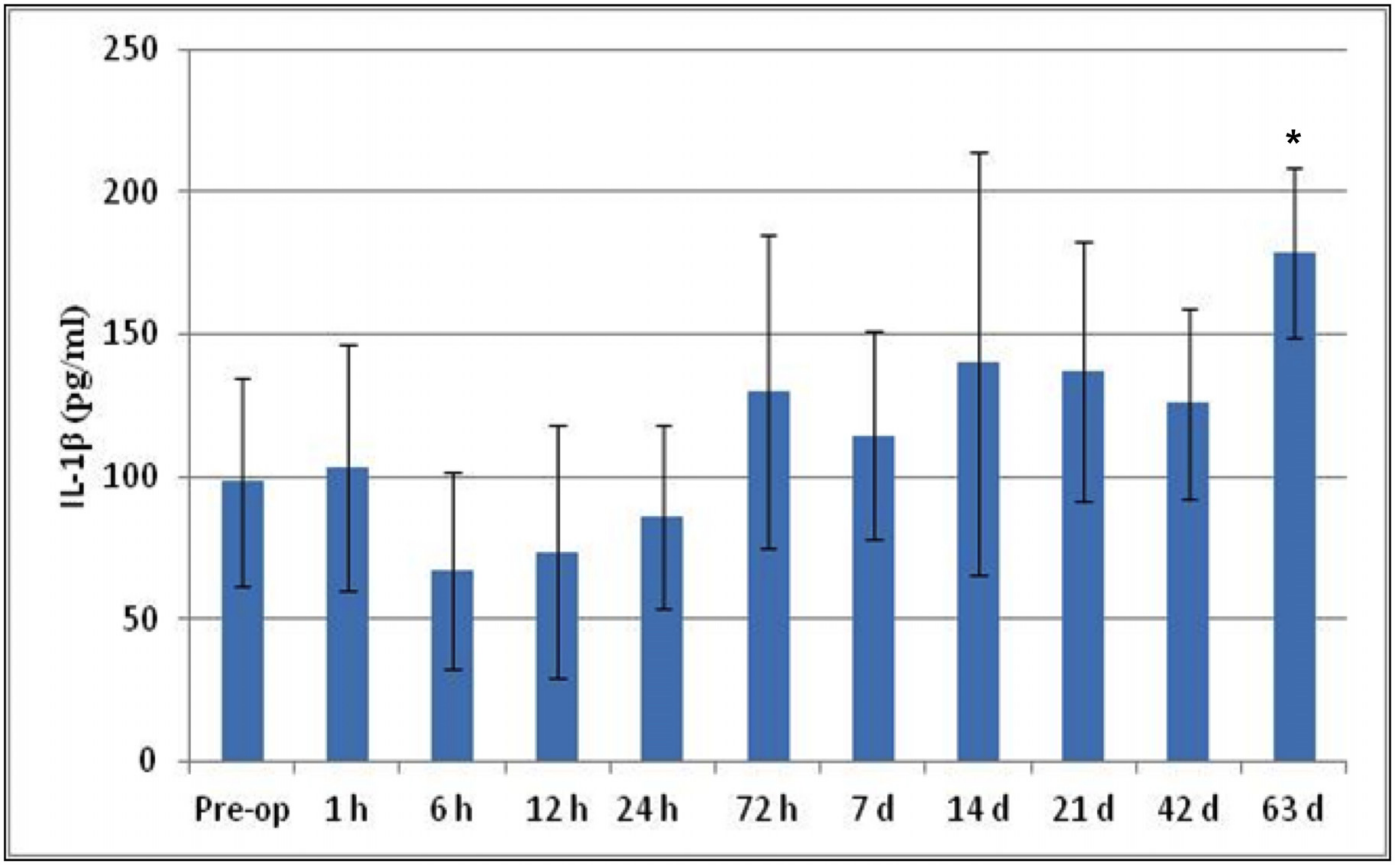
Serological cytokine expression for IL-1β over the time course. IL-1β levels were detected at all the allocated time points with a significant difference between pre-op and day 63 (*p<0.05).

**Fig 7:**
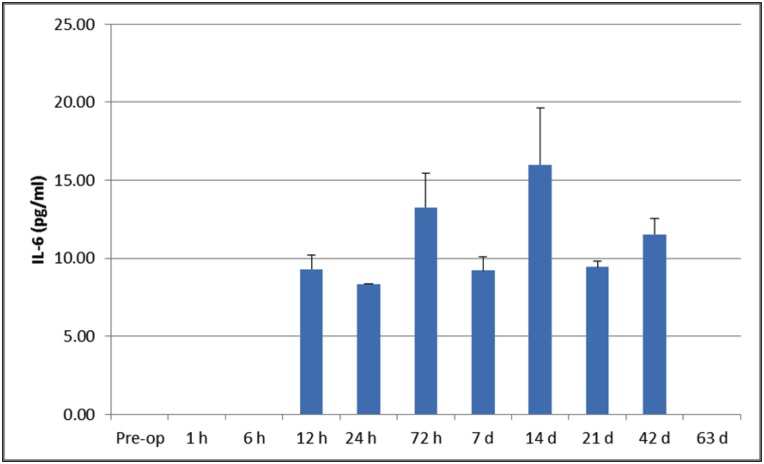
Serological cytokine expression for IL-6 over the time course. IL-6 levels were only detected from 12hrs to 42 days.

IL-1β levels were detected at all the allocated time points with a significant difference between pre-op and day 63 expression (p<0.05), however IL-6 levels were only detected from 12hrs to 42 days. These are shown in Figs 6 and 7. The levels of serum cytokine for TNF-α in all cases, at all the time points, was below the sample detection level (data not shown).

### Tissue sample harvesting and Vancomycin / Tobramycin levels

**Fig 8:**
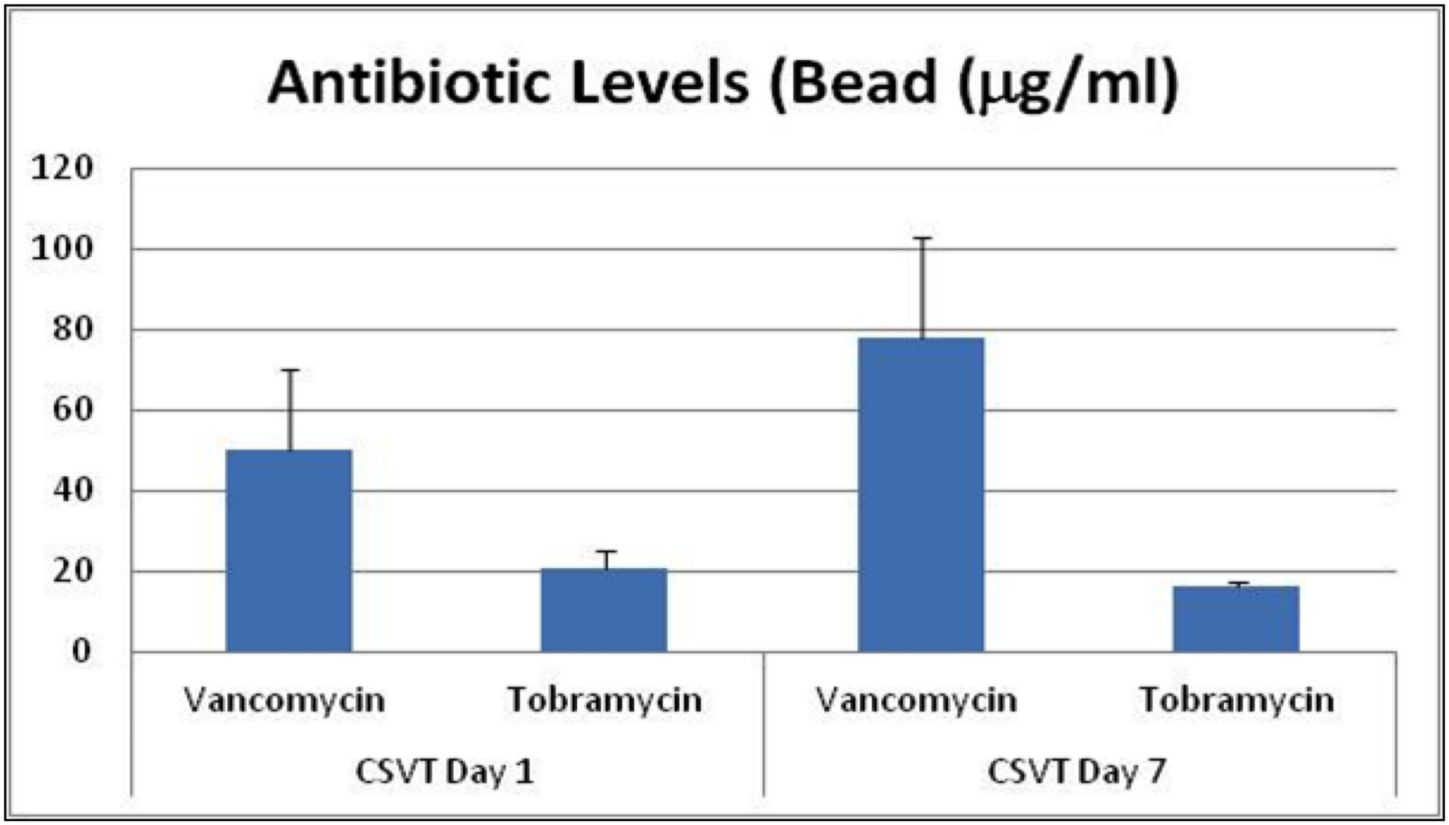
Graphical representation of antibiotic levels within beads. Measured vancomycin and tobramycin levels within the remnant beads found at the implantation site at 24 hours and day 7.

During harvest the surgical sites were carefully dissected and examined for the presence of any residual calcium sulfate beads. A portion (0.1g) of any residual calcium sulfate bead present was harvested. Residual beads were only found in animals allocated for 24 hours and day 7 time points. Vancomycin and tobramycin levels in the samples per gram of material (CSVT) were determined. Results are represented in Fig 8.

Muscle samples (1x1cm) from the implantation sites were harvested and then minced and air dried. The local concentration of vancomycin and tobramycin in the muscle sample was measured using a standard antibody assay. Antibiotic was below the level of detection in any samples analysed for 24 hours and day 7 harvested samples, thus later time points were not examined.

### Radiography

Posterior anterior radiographs were taken as per schedule at the time of harvest and post operatively. Radiographs were graded from 0 (81–100% implant remaining) to a grade of 4 (0–20% implant remaining).

**Fig 9:**
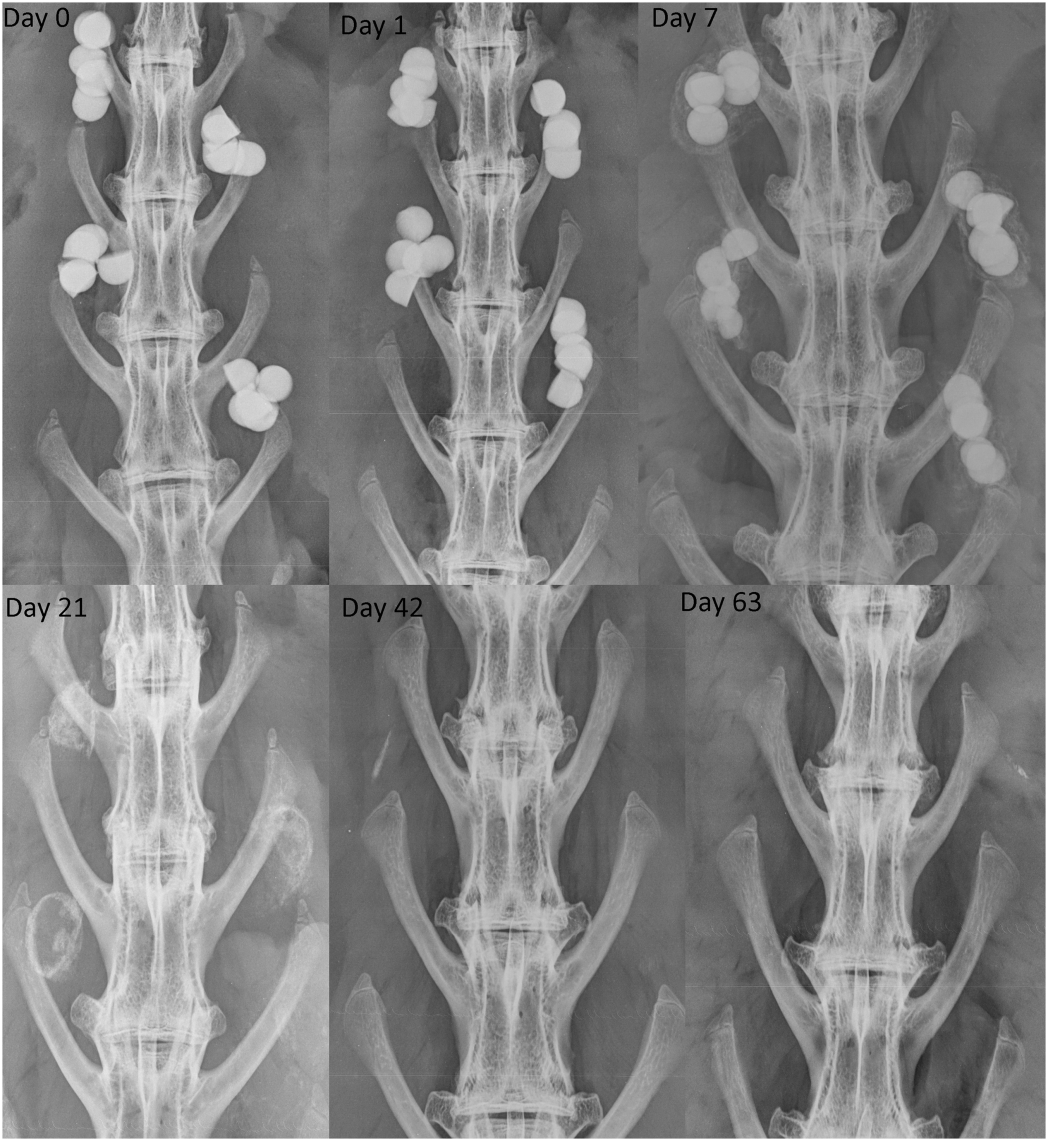
Representative Radiography Panel. Beads were present at Days 0, 1 and 7. Complete absorption was noted by day 21.

**Fig 10:**
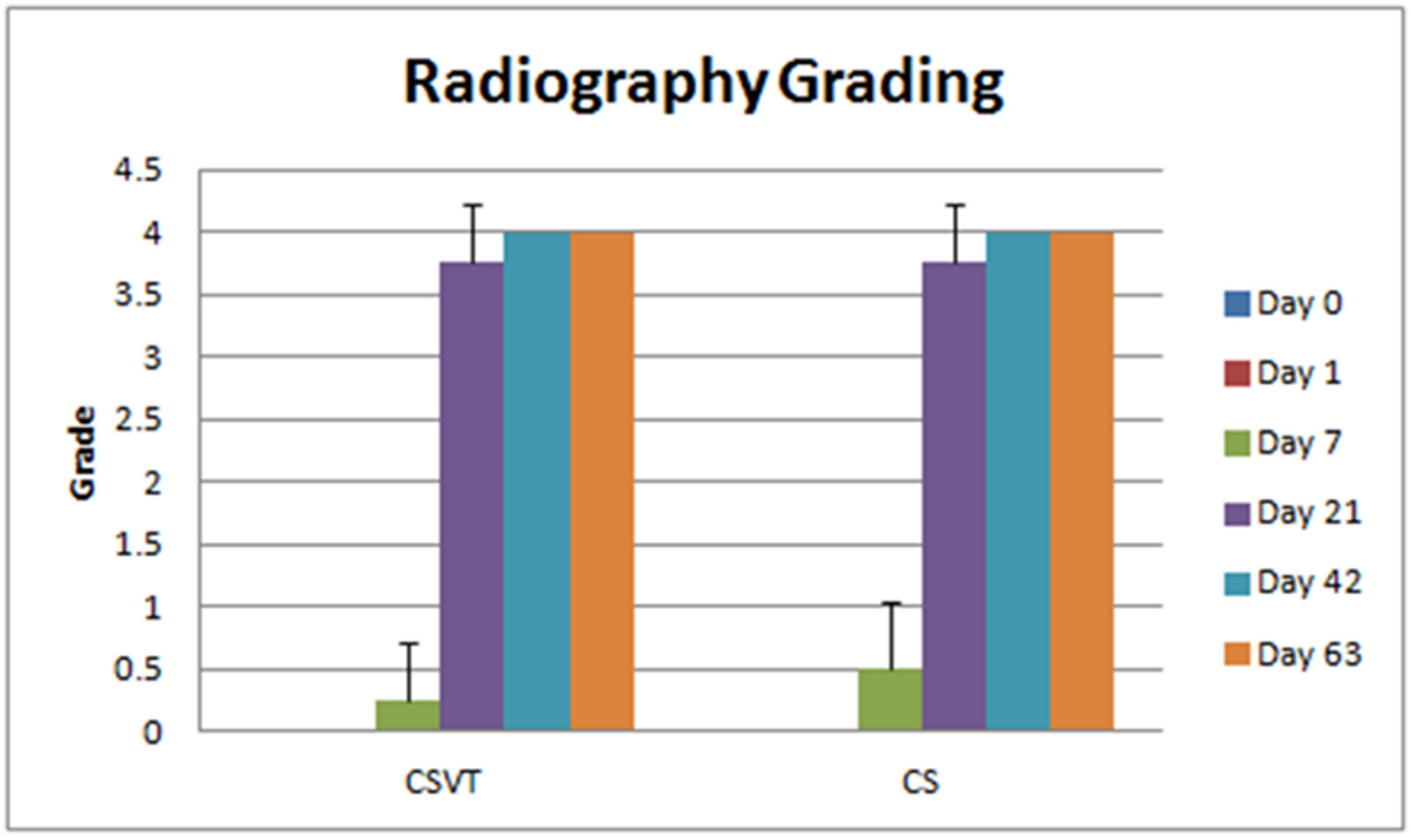
Summary of mean Faxitron resorption grading for CSVT and CS groups at each time point. No difference between groups was observed.

CS and CSVT beads were apparent in day 0, 24 hours, and day 7 radiographs. Some softening of the beads as well as some areas of lucency was observed on day 7. Complete absorption of all beads was confirmed by day 21, and only scarce areas of bead consolidation were noticeable. Beads were not evident at the 42 and 63 day radiographs (Fig 9). Radiographic grading revealed the same absorption patterns for CSVT and CS beads as demonstrated in Fig 10. Migration of the implanted beads was not evident from the radiographs.

A faint halo of material was noted in the muscle tissue around both CS and CSVT beads on the radiographs at 24 hours and 7 days. The halo remained at 21 days despite complete absorption of the beads. However, this halo of material was no longer present on the 42 and 63 day radiographs.

### Micro Computed Tomography

**Fig 11:**
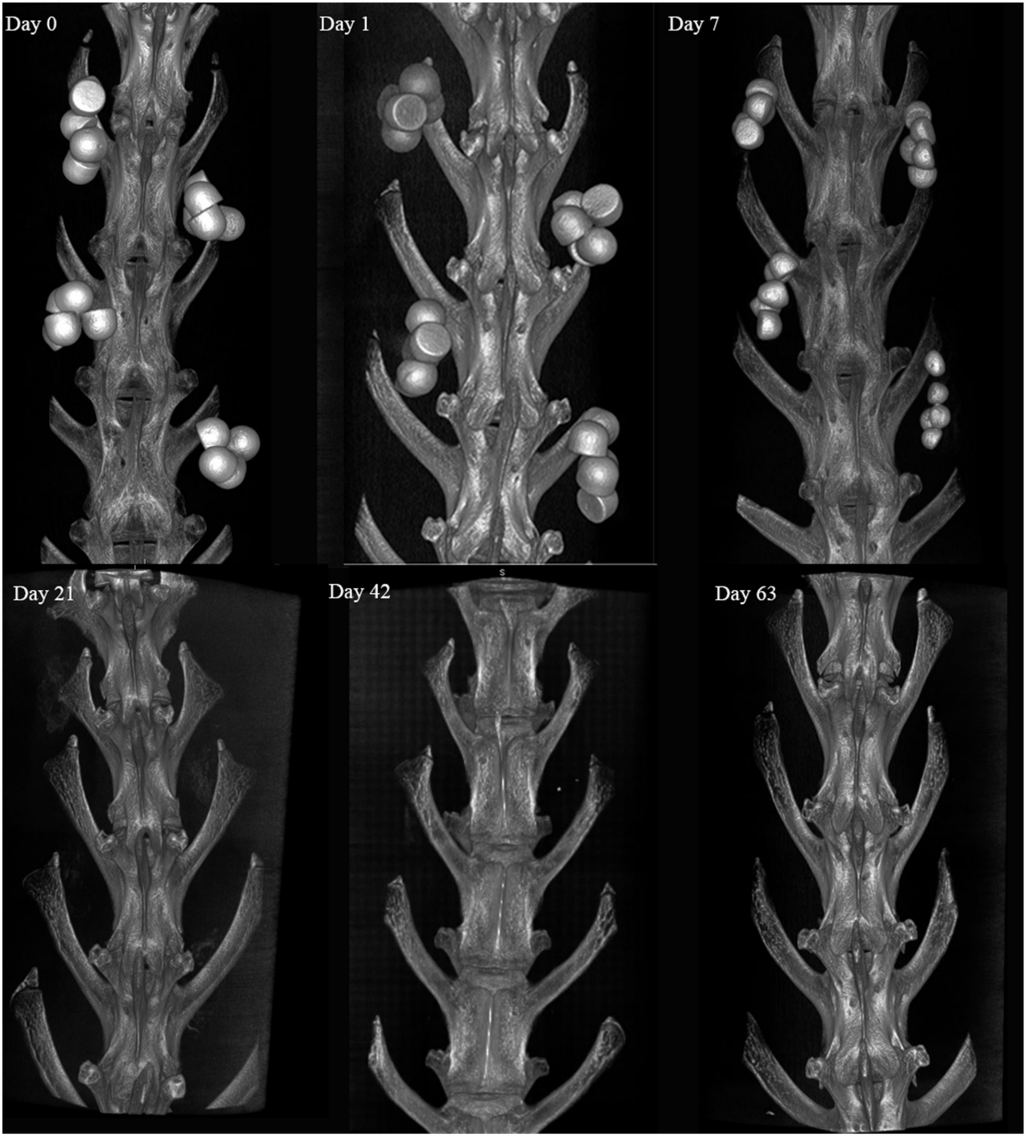
Representative 3D MicroCT Panel. MicroCT demonstrating the absorption pattern for the implanted materials at Days 0, 1, 7, 21, 42 and 63 days.

**Fig 12:**
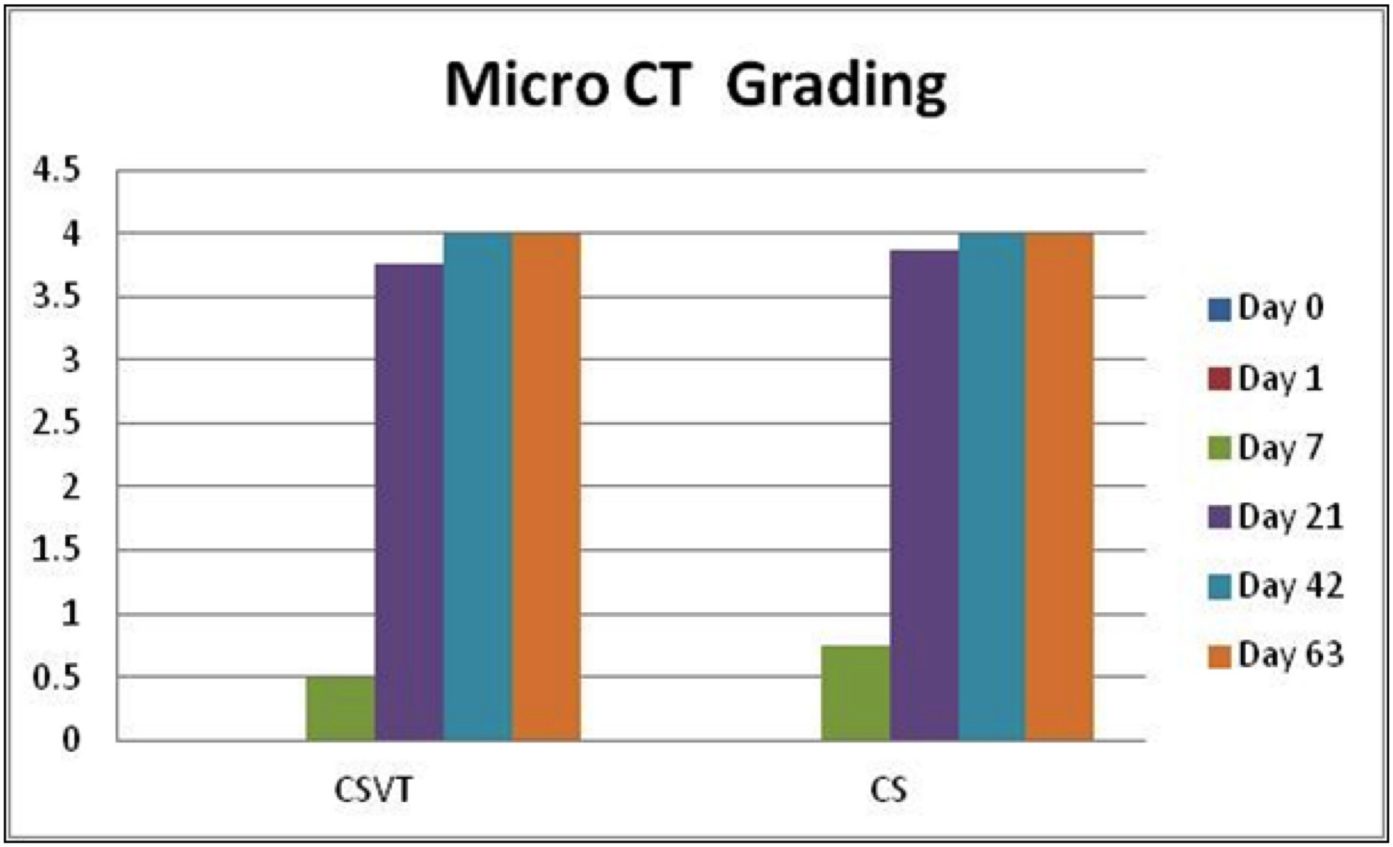
Summary of mean μCT resorption grading for CSVT and CS groups at each time point. No difference between groups was observed.

μCT provided an assessment of bead absorption in the axial, sagittal, and coronal anatomical planes and confirmed the radiographic findings. All beads were absorbed by the 21 day time point (Fig 11). No adverse reactions to the implanted materials were noted. μCT grading revealed similar absorption patterns for both implanted materials as demonstrated in Fig 12.

### Histology

#### Qualitative analysis

For implantation sites at day 0, no local reaction to the implant material was noted at any of the implantation sites. For implantation sites at 24 hours, the reaction to CS and CSVT was identical in nature and magnitude, with lymphocytes and red blood cells observed at the implant /host tissue interface. At day 7, once again, the reaction to both materials was identical in nature and magnitude. Lymphocytes and red blood cells were observed at the implant /host tissue interface. In addition some multinucleated cells were present and some infiltration of fibrous tissue was observed. At days 21, 42 and 63, the response to both materials was identical in nature and magnitude, all with lymphocytes and red blood cells at the implant /host tissue interface, and multinucleated cells present. The materials had completely resorbed and were replaced by fibrous tissue.

### Immunohistochemistry

**Fig 13:**
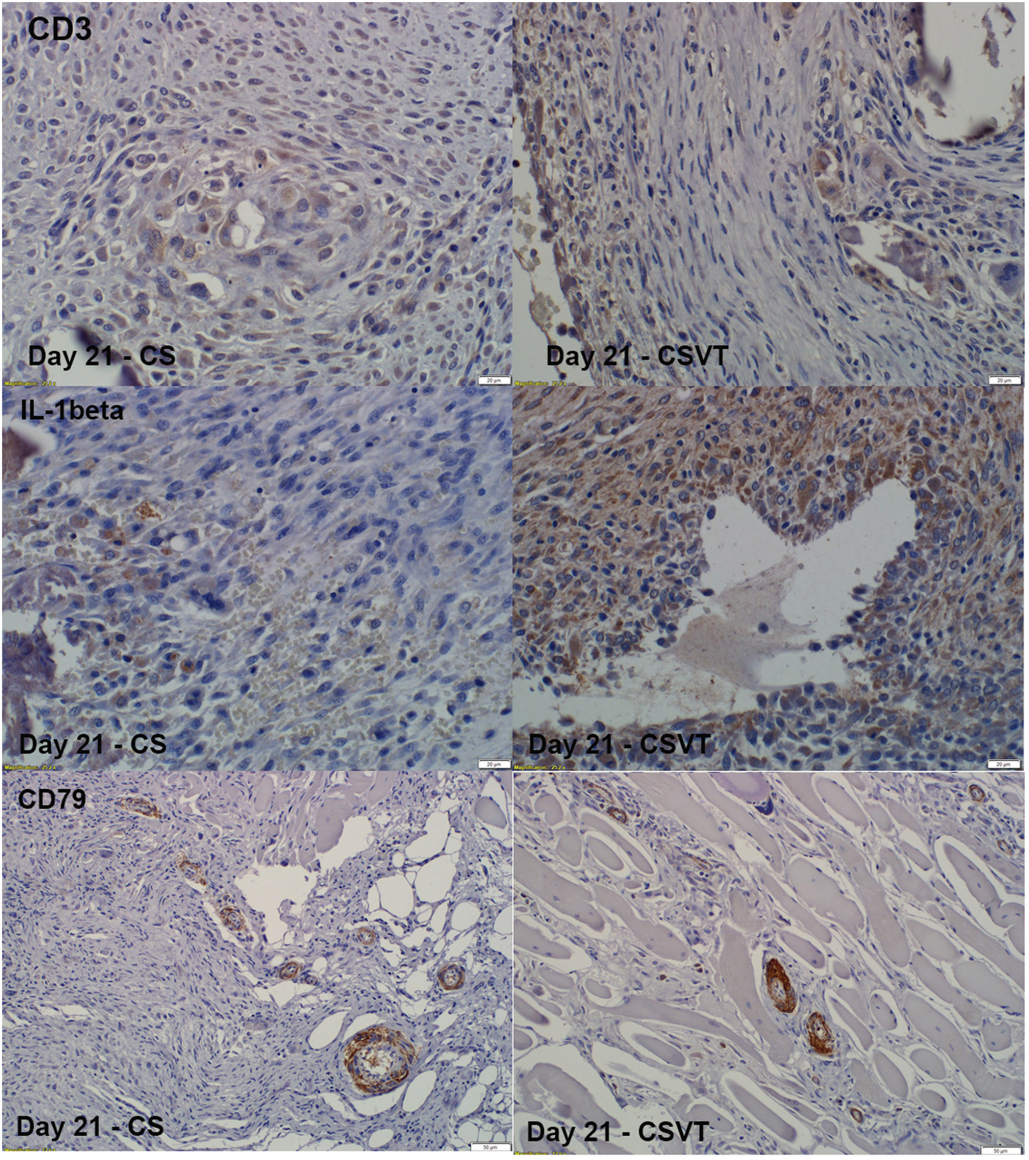
Immunohistochemistry for CD3, IL-1β and CD79 expression. Some expression for these markers were found in the CS and CSVT groups at 21 days (positive signal by brown staining). The same overall expression to the materials was seen at the other time points.

Immunhistochemistry demonstrated protein expression for markers of inflammation (IL1-Beta, CD3, CD79 and IL-6). Some expression for these markers were found in the CS as well as the CSVT groups at days 7, 21, 42 and 63 and was similar between CS and CSVT at each time point. The overall *in-vivo* response to the materials was the same (Fig 13).

**Fig 14:**
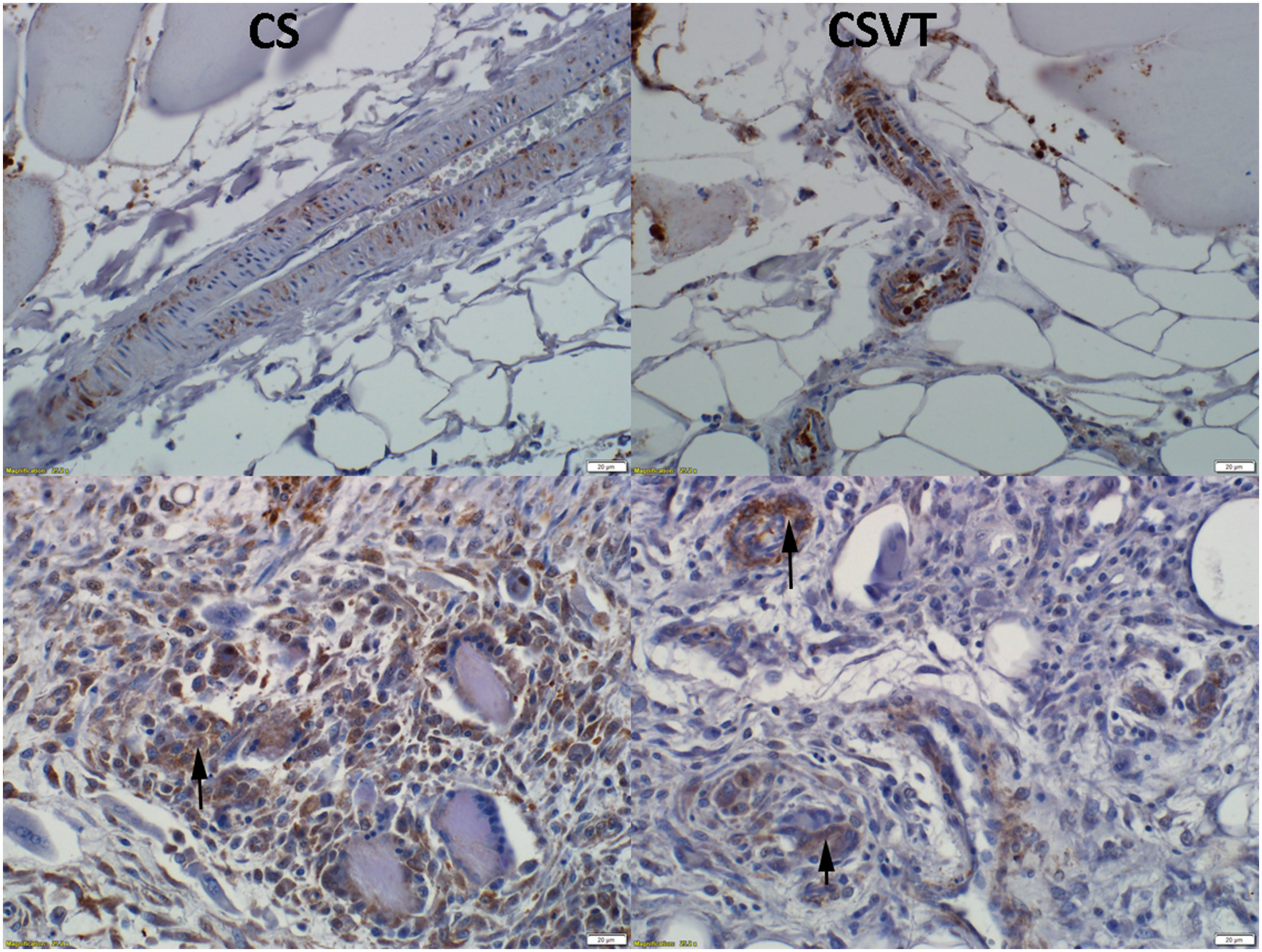
Immunohistochemistry for IL-6 expression. Expression for IL-6 was noted at day 1 (positive signal by brown staining) in adjacent blood vessels as well as at day 7 in endothelial cells and multi-nucleated cells (macrophage lineage) (arrows).

IL-6 was expressed at day 1 in adjacent blood vessels. Expression was noted at day 7 in endothelial cells as well as multi-nucleated cells (macrophage lineage) (Fig 14).

## Discussion

Dead space can be defined as a space remaining in a wound after surgical closure. It is frequently a result of the removal of implants and the extensive debridement of necrotic tissue following joint revision surgery. If the dead space is not eradicated, it quickly fills with hematoma and wound exudate immediately following surgery, which provides ideal conditions for bacterial growth for residual pathogens present in the surgical site. Once bacteria are established in this space, infection is difficult to eradicate due to the limited ability of parenterally administered antibiotics to penetrate the space as it is not vascularised. Therefore it is paramount that dead space is minimised. This can be achieved with the transfer of muscle flaps [33–37] or filling the space with a device or implant material which minimises the potential for hematoma formation. PMMA is frequently used to perform this function, either in the form of a spacer [8, 9, 38] in joint revision surgery, or beads [5, 39] which are frequently used in cases of osteomyelitis. However, as discussed previously, PMMA does have some drawbacks which have resulted in clinicians seeking alternative, resorbable materials for dead space management. One such material is calcium sulfate, with recent literature discussing its application in combination with antibiotics [12, 40, 41], including its use in infected soft tissue sites [42].

A number of animal models have been published which enable the evaluation of implant materials to fill voids in bone following osteomyelitis [24, 43–45], however no animal models have been published to evaluate the efficacy of an implant material for the management of dead space in soft tissue. This study describes a novel animal model to investigate dead-space in soft tissue and evaluate implant material for its management.

The model offers a number of advantages. Firstly, it allows multiple surgical sites per animal which makes the comparison of host response to device / material variations possible in the same animal, and allows fewer animals to be studied considering the internal controls. Also, after scaling up to human clinical subjects, the size of the intramuscular site is comparable to potential dead space observed in clinical scenarios following extensive debridement. Finally, the model is large enough to easily allow a comprehensive range of analysis techniques, including radiographic analysis (standard radiographs and μCT), multiple blood serum analysis, tissue analysis, and complete histological analysis to characterise and quantify the host response to the implant material under test.

The absorbable calcium sulfate material chosen to evaluate the model has been reported previously in literature as a means of dead space management in joint revision surgery [46–51] and infected soft tissue sites [42, 52]. The model evaluated the calcium sulfate alone (CS) and in combination with two antibiotics, vancomycin and tobramycin (CSVT). The choice and quantity of the antibiotics chosen reflects those reported previously in the literature [46, 47, 49–51]. The choice of antibiotic for incorporation into a device for dead space management must be carefully considered if it is to offer optimal protection from bacterial colonisation.

Vancomycin is a glycopeptide antibiotic used in the prophylaxis and treatment of infections caused by Gram-positive bacteria. Tobramycin is an aminoglycoside antibiotic used to treat various types of bacterial infections. The combination of vancomycin with tobramycin is favourable due to the synergistic activity of the two antibiotics with respect to Gram-negative and Gram-positive bacterial coverage [53, 54]. Vancomycin shows very poor systemic tissue penetration [55, 56] which has been considered a disadvantage in intravenous application [57, 58]. However this could be an advantage in its use to protect an implant from microbial colonization, since there may be reduced penetration from the implant site into the vascular system, keeping vancomycin levels high adjacent to the implant and systemic levels low. The current study showed a lack of detectable serum levels of vancomycin keeping with a poor systemic penetration, and with clinical observations.

Elution characteristics of tobramycin impregnated calcium sulfate beads have previously been evaluated [59–61]. Systemic concentrations achieved even after experimentally implanting 1.8 times the maximum prescribed human dose to dogs were undetectable after 24 hours, with no signs of systemic toxicity [60]. The systemic levels of tobramycin measured in the current study are consistent with this observation, with systemic tobramycin levels subsiding in the majority of animals within 24 hours.

Analysis of residual CSVT beads harvested at 7 and 21 days contained some residual vancomycin and tobramycin, with a higher quantity of vancomycin present. As a result of the large standard deviation in the measured levels for both antibiotics, a comparison of relative levels of antibiotic present in residual beads with the original ratio in the beads as prepared is of limited relevance. When the local concentration of vancomycin and tobramycin in the harvested muscle samples was measured, levels were below the level of detection in all samples analysed for 24 hours and day 7 post implantation. This contradiction may be a result of a lack of antibiotic binding to the harvested muscle tissue and warrants further investigation.

The histological response to the implanted materials was in keeping with the expected *in-vivo* response to the implantation of a calcium sulfate which is a well-tolerated implant material, with the host tissue response graded as mild to moderate. This is an interesting observation when considering the nature of the implant materials and their constituents. Tobramycin sulfate is safe for intramuscular administration at the correct dosage and is subsequently rapidly absorbed. In contrast, vancomycin hydrochloride solutions have a low pH (2.8–4.5), and are irritating to tissue, caused drug fever, pain and possible necrosis. As such it is not administered intramuscularly, with intravenous administration the recommended route. Stimulan is produced synthetically from pharmaceutical purity reagents. It has a neutral to mildly alkaline physiological pH. This is different to other CS which are produced from gypsum rock and tends to have an acidic pH. Clinical studies using Stimulan with vancomycin and tobramycin show very low levels IL-1β plays a role in tissue repair and the elevated levels at day 63 are consistent with the histological response of fibrous tissue at the sites were the material had completely resorbed [46].

The host tissue response to both CS and CSVT was the same, indicating no additional adverse local tissue response to the presence of both vancomycin and tobramycin in the CSVT beads. The potential additional tissue irritation that could be caused by the presence of vancomycin was not evident. This may be due to the low levels of vancomycin hydrochloride present in the CSVT beads, or as a result of some change to a characteristics of the antibiotic that elicit an adverse reaction. A possible cause is a buffering of the acidic pH of vancomycin hydrochloride as a result of its combination with calcium sulfate.

The study had a number of limitations. Although a dead space is typically created by resection of a volume of tissue in a clinical situation, in our model we retracted the muscle and created the dead space. Cutting out a piece of muscle to elicit a blood filled muscular/soft tissue defect would have elicited a different type of tissue response. No sham procedure was employed in the model to determine the effect of the creation of an unfilled intramuscular site. It was felt that this would have been of limited use, as the intramuscular site would have closed with the formation of dead space if no implant material was placed. The model as described is not an infection model. Therefore it was not possible to determine the in-vivo efficacy of the antibiotic in the CSVT implant on protecting the material from bacterial colonisation, although this has recently been shown in-vitro [62]. The introduction of soft tissue infection to this model would improve its use in determining the efficacy of devices for dead space management and any resulting reduction in re-infection rates as a result. Due to the added procedural complexity animal urine was not collected and analysed in the study. This may have provided a fuller picture of the pharmacokinetics of the antibiotics combined with the CSVT implant.

## Conclusion

The model was found to be a convenient and effective model for the assessment of implant materials for management of dead space in muscle tissue. The two materials tested were effective in resolving the surgically created dead space, and did not elicit any unexpected adverse host response.
